# Rx for Addiction and Medication Safety (RAMS-PEER): Evaluation of an Education and Peer Program on Opioid Misuse

**DOI:** 10.5888/pcd17.190380

**Published:** 2020-05-21

**Authors:** Tianyu Sun, Ashley L. Buchanan, Jeffrey P. Bratberg, Emily Patry, Kelly L. Matson

**Affiliations:** 1 University of Rhode Island, Department of Pharmacy Practice, Kingston, Rhode Island

## Abstract

The Rx (prescription) for Addiction and Medication Safety (RAMS) program was developed during the 2017 through 2018 academic year to educate students from 6 selected Rhode Island public high schools about opioid misuse, overdose, and recovery. During 2016, 3 schools participated in the RAMS program and returned for RAMS-PEER in 2017; 3 schools were newly recruited in 2016. Tenth graders returned from schools that participated during RAMS in 2016, and all ninth graders were new. Our study’s aim was to evaluate the overall effect and spillover benefit of the RAMS-PEER intervention from tenth to ninth graders by surveying students both before and after the education program. Survey questions were modified from the 2015 Youth Risk Behavior Survey and the 2015 Ontario Study Survey. Student responses were matched for preintervention and postintervention analysis using a unique identifier. We observed an improvement in knowledge of opioid misuse; however, we found no evidence of a significant spillover benefit.

**Summary**

**What is already known on this topic?**

Rhode Island adolescents had among the highest estimated prevalence rates of illicit drug use from 2015 to 2016.

**What is added by this report?**

The Rx (prescription) for Addiction and Medication Safety program was developed to deliver opioid misuse education for Rhode Island public high schools to reverse the trend of illicit drug use. Results found student knowledge of opioid misuse and use disorder improved following a pharmacy-implemented intervention. However, spillover benefits were not observed, indicating that consistent program delivery may be needed.

**What are the implications for public health practice?**

Our study provides evidence of an effective adolescent opioid misuse awareness program and, perhaps, a foundation for a statewide opioid misuse educational program within Rhode Island public schools.

## Introduction

The United States is facing an unprecedented crisis of drug overdose. More than 70,200 people died from an overdose during 2017 alone. From the 70,200 deaths, 47,600 (68%) were overdoses from all opioids, and 17,029 (24%) were overdoses from prescription opioids (1). In Rhode Island, the opioid overdose death rate was 26.9 per 100,000 population and ranked tenth in the United States in 2017. RI adolescents were especially at high risk of substance misuse. Based on the 2015–2016 National Survey on Drug Use and Health, RI had one of the highest estimated percentages of adolescents (aged 12 to 17 y) who used illicit drugs, 12.5% (95% confidence interval [CI], 10.18%–15.19%), compared with the national average of 8.3% (95% CI, 7.98%–8.71%) (2).

To prevent opioid misuse and overdose among youth, we implemented the Rx for Addiction and Medication Safety (RAMS) program. Developed by the University of Rhode Island (URI) College of Pharmacy, RAMS was an opioid misuse prevention education program delivered by trained pharmacy students for freshmen students at 8 public high schools in Rhode Island during the 2016 through 2017 academic year. Of students surveyed, 33 (7%) significantly improved their identification of opioid misuse, 19% improved knowledge of opioid withdrawal symptoms, 14% improved knowledge of the need for treatment referral, and 28% improved knowledge of naloxone administration (3). The PEER program was developed as a 1-hour, online, supplemental curriculum for RAMS. We implemented the RAMS-PEER program during the subsequent 2017 through 2018 academic year to boost learning effects and evaluate potential spillover benefit of peer education. Three of 8 schools from the previous academic year returned to the program, and 3 new public high schools began participation. We delivered the booster course only to tenth-grade students from the returning schools who had participated in the RAMS program during the previous year. The full 3-hour RAMS curriculum was then delivered to ninth graders in all 6 schools.

## Purpose and Objectives

Our study aimed to evaluate the overall effect and possible spillover benefit of the RAMS-PEER intervention among high school students, specifically, to determine if tenth graders participating in the prior year shared their knowledge with incoming ninth graders (4–6). Leveraging spillover mechanisms to sustainably scale up and deliver the RAMS intervention is necessary, given the often resource-constrained setting of public-school systems. Recent evidence points to stronger influences of peer-to-peer education for risk reduction, as compared with provider-to-peer, and we anticipated this resonance might be stronger among adolescent peers (5,7). We conclude with a discussion of next steps to evaluate spillover of the RAMS-PEER intervention.

## Intervention Approach

RAMS is a 3-hour, on-site, school-based curriculum, providing 3 to 4 interactive educational sessions. Sessions include medication safety, signs, symptoms, and risk factors for opioid misuse and withdrawal; opioid overdose identification and response; and local treatment and recovery resources. Implementation of RAMS has been described previously (3). The RAMS-PEER program is a booster curriculum for RAMS and designed as an online supplement. Seven, 5-minute videos were created for RAMS-PEER to highlight RAMS education, and the curriculum includes facilitator guides for instructors’ in-class discussions. All RAMS-PEER materials were available on the RAMS-PEER website (www.ramspeer.com) during the program’s implementation. Returning tenth graders who had received the RAMS curriculum in the first year were instructed to view videos either in school or in their spare time. A discussion led by high-school faculty was mandatory after each booster video. Once completed, URI pharmacy students delivered the primary RAMS program to ninth graders at both new and returning schools.

## Evaluation Methods

We conducted a pre-post comparison study to measure an initial RAMS-PEER intervention on student knowledge, perceptions, risk, and protective behaviors related to opioid misuse. Our study population included students entering the ninth and tenth grades during the 2017 through 2018 academic year. Students voluntarily participated in 2 confidential surveys answering questions by using SurveyMonkey (SVMK, Inc). Before program delivery (preintervention), ninth graders completed a 20-minute survey that included risk and protective factors for substance misuse; past 30 days nonmedical use of opioids, alcohol use, and use of other substances; students’ perception of risk or harm from prescription drugs and prescription drug overdose; awareness of resources and treatment of substance misuse; proper disposal and storage of prescription medications; and how to obtain and administer naloxone. These items were modified from the 2015 Youth Risk Behavior Survey from the Centers for Disease Control and Prevention and the 2015 Ontario Study Survey from the Canadian Centre for Addiction and Mental Health and identical to surveys administered in the RAMS first-year program (3,8,9). The survey also collected student demographic information, such as age, race/ethnicity, and sex, as well as home environment, social media use, self-reported mental health, academic assessment, and substance use. The same survey was administrated again 1 to 2 months after the RAMS-PEER curriculum was completed among the ninth and tenth graders (postintervention). In this analysis, the intervention was a booster for tenth graders from returning schools and a full curriculum for ninth graders from all 6 schools. All postsurveys were administered after all students received the full intervention. Each student response was matched for preintervention and postintervention analysis using a unique identifier.

Descriptive statistics, including means and proportions, were used to report participant demographics and other characteristics. We used the χ^2^ test to conduct cross-sectional and paired comparisons between outcomes at the 6 schools at preintervention and postintervention. Because the matched sample had missing values (n = 129, 40%), a multiple imputation approach was used with discriminant function for categorical variables to impute missing values. We used the fully conditional specification method to impute the missing data. Multiple imputation was performed on combined preintervention and postintervention data to generate 30 data sets with complete information, assuming a multivariate normal distribution and that missing data depended only on observed covariates (10,11). CIs for imputation results were based on standard errors using Rubin’s estimator of variance (12). To assess if ninth grade students in returning schools had an associated improvement in knowledge due to the educational program (RAMS) and the booster (PEER), as compared with ninth grade students in new schools who received only RAMS, we fit a logistic model with binary versions of each outcome (responses to knowledge questions were identified as either correct or incorrect; 5-level confidence questions were dichotomized into 2 groups (eg, none of your closest friends use pain relief pills versus at least some). In the models for matched precomparisons and postcomparisons, each student served as their own control; therefore, time-invariant confounding was subtracted by individual level differencing, and secular trends were less of a concern, short of a follow-up period. We also included the confounders of age, sex, race, grade, access to pain relief, and mental health (significance set at *P* < .20) in the logistic model evaluating the association of school type on outcomes. Statistical analysis was completed using SAS Version 9.4 (SAS Institute, Inc), and all tests were 2-sided at *P* < .05 significance.

To evaluate the possible spillover and peer education benefit from tenth to ninth graders in returning schools, we calculated the adjusted odds ratios (aORs) of correct responses or improvement in knowledge among ninth graders in returning schools versus ninth graders in new schools. Improvement in knowledge was defined as having correct answers in both the presurvey and the postsurvey, or having incorrect answers in presurvey and correct answers in the postsurvey. 

## Results

Of ninth grade students in 2017 through 2018, 1,030 participated in the preintervention survey, and 439 participated in the postintervention survey. We matched 321 students with both preintervention and postintervention surveys for the year. Most ninth graders from the 6 high schools were aged 14 or 15 years (n = 1,013, 98.3%), 823 were white (79.9%), and 908 (88.2%) earned nearly all A and B grades. Of the 1,030 students, 715 (69.4%) reported good mental health status, with no feelings of worthlessness (n = 594, 57.7%) or hopelessness (n = 622, 60.4%). Additionally, 707 students found it difficult or did not know how to obtain pain relief medication (68.6%) or medication to treat attention deficit hyperactivity disorder without prescription (n = 759, 73.7%) (Table 1).

**Table 1:** RAMS-PEER Intervention Surveys and Matched Respondent Demographic Characteristics Among Ninth Grade Students, Rhode Island, 2017

Characteristic	RAMS-PEER 2017 Presurvey, N = 1030, n (%)^a^	RAMS-PEER 2017 Matched Presurvey and Postsurvey, N = 321, n (%)^a^
**Sex**		
Female	493 (47.9)	190 (59.2)
Male	518 (50.3)	128 (39.9)
Other	19 (1.8)	3 (0.9)
**Age, y**		
14	551 (53.5)	127 (39.6)
15	462 (44.9)	188 (58.6)
16	11 (1.1)	5 (1.6)
17	6 (0.6)	1 (0.3)
**Race**		
White	823 (79.9)	271 (84.4)
Other	207 (20.1)	50 (15.6)
**Grades**		
B or better	908 (88.2)	299 (93.2)
Less than B	122 (11.8)	22 (6.9)
**Daily social media use, h**		
<2	349 (33.9)	109 (34.0)
2–4	481 (46.7)	148 (46.1)
>5	199 (19.3)	64 (19.9)
Missing	1 (0.1)	0
**How easy or difficult would it be for you to get pain relief pills without going to a doctor?**		
Easy	269 (26.1)	88 (27.4)
Difficult	400 (38.8)	142 (44.2)
I don't know	307 (29.8)	78 (24.3)
Missing	54 (5.2)	13 (4.0)
**How easy or difficult would it be for you to ADHD medications without going to a doctor?**		
Easy	205 (19.9)	74 (23.1)
Difficult	419 (40.7)	146 (45.5)
I don't know	340 (33.0)	86 (26.8)
Missing	66 (6.4)	15 (4.7)
**Self-reported emotional health**		
Good	715 (69.4)	219 (68.2)
Poor	63 (6.1)	19 (5.9)
Missing	252 (24.5)	83 (25.9)
**Self-reported hopeless feeling**		
Little or no	594 (57.7)	169 (52.7)
Some time or more	178 (17.3)	67 (20.9)
Missing	258 (25.1)	85 (26.5)
**Self-reported worthless feeling**		
Little or no	622 (60.4)	186 (57.9)
Some time or more	150 (14.6)	50 (15.6)
Missing	258 (25.1)	85 (26.5)

Abbreviations: ADHD, attention deficit hyperactivity disorder; RAMS, Rx for Addiction and Medication Safety.

a Values of polytomous variables might not sum to 100% due to rounding.

After the RAMS-PEER program, during the 2017 through 2018 academic year, ninth grade student knowledge of opioid misuse improved. The percentage of correct answers increased significantly from preintervention to postintervention for questions on knowledge of opioid misuse (from 76.2% correct to 84.4% correct), and perceptions of people who use drugs (from 27.4% correct to 33.9% correct) (Table 2).

**Table 2:** Improved Knowledge Comparison Among Ninth Grade Students From Returning Schools Versus New Schools, Rhode Island, Academic Year 2017–2018

Questions (Q) and Dichotomized Answers	2017 Presurvey N =1,030, n (%)	2017 Postsurvey N = 415, n (%)	*P* Value^a^	Matched 2017 Presurvey N = 321, n (%)	Matched 2017 Postsurvey N = 321, n (%)	*P* Value	Adjusted Odds Ratios (95% Confidence Interval)
**Q93 Addiction is a chronic brain disease or disorder.^b^**							
Correct	591 (79.3)	284 (83.5)	.10	191 (82.0)	232 (86.6)	.15	1.08 (0.63–1.83)
**Q93 Drug misuse is accepting prescription medication from a friend.^b^**							
Correct	568 (76.2)	287 (84.4)	.01	181 (77.7)	230 (85.8)	.01	0.72 (0.44–1.18)
**Q93 Drug misuse is use of a prescription medication is exceeding the recommended dose.^b^**							
Correct	653 (87.7)	305 (89.7)	.33	208 (89.3)	245 (91.4)	.41	0.85 (0.48–1.51)
**Q93 Nonmedical use is using a prescription medication without a prescription.^b^**							
Correct	634 (85.1)	299 (87.9)	.21	209 (89.7)	240 (89.6)	.95	1.77 (0.86–3.63)
**Q100 Drug users are responsible for their addiction.^c^**							
Correct	202 (27.4)	114 (33.9)	.03	67 (29.0)	91 (34.2)	.21	0.93 (0.60–1.44)
**Q100 Drug users can stop using drugs whenever they want to.^c^**							
Correct	557 (75.5)	272 (81.0)	.05	182 (78.8)	22 (83.1)	.22	1.13 (0.70–1.82)
**Q100 People use drugs to avoid dealing with their own inadequacies.^c^**							
Correct	128 (17.3)	80 (23.8)	.01	40 (17.3)	53 (19.9)	.45	1.26 (0.79–1.98)
**Q100 Drug users have weak characters^c^**							
Correct	442 (59.9)	213 (63.4)	.28	147 (63.6)	17 (63.9)	.95	0.97 (0.65–1.43)
**Q76 Feel so depressed (sad) that nothing could cheer you up?^d^**							
None	489 (63.3)	204 (58.1)	.09	138 (58.5)	16 (58.8)	.93	1.16 (0.75–1.81)
A little, sometime, most time, or all the time	283 (36.7)	147 (41.9)	NA	98 (41.5)	11 (41.2)	NA	NA
**Q78 In the last 4 weeks, did you feel that you were under any stress, strain, or pressure?^d^**							
Not at all	101 (13.1)	47 (13.4)	.89	20 (8.5)	34 (12.3)	.16	1.96 (0.83–4.61)
A little, some, a lot, or more than I could take	671 (86.9)	304 (86.6)	NA	216 (91.5)	24 (87.7)	NA	NA
**Q83 How many of your CLOSEST friends use pain relief pills such as Percocet, Tylenol #3, Vicodin, Oxycodone?^e^**							
None	540 (70.8)	242 (69.3)	.628	160 (68.1)	19 (69.2)	.78	0.92 (0.64–1.39)
Some, most, all, or don’t know	223 (29.2)	107 (30.7)	NA	75 (31.9)	85 (30.8)	NA	NA
**Q97 My parent(s) showed me affection.^f^**							
Always or frequently	640 (86.7)	284 (84.5)	.34	201 (87.0)	22 (85.7)	.67	1.38 (0.76–2.52)
Sometimes or rarely	98 (13.3)	52 (15.5)	NA	30 (13.0)	38 (14.3)	NA	NA

a *P* value was calculated by using χ^2^ test.

b 285 Missing values in 2017 presurvey, 75 in 2017 postsurvey, 88 in matched presurvey, and 53 in matched postsurvey.

c 292 Missing values in 2017 presurvey, 79 in 2017 postsurvey, 90 in matched presurvey, and 55 in matched postsurvey.

d 258 Missing values in 2017 presurvey, 64 in 2017 postsurvey, 85 in matched presurvey, and 44 in matched postsurvey.

e 267 Missing values in 2017 presurvey, 66 in 2017 postsurvey, 86 in matched presurvey, and 45 in matched postsurvey.

f 292 Missing values in 2017 presurvey, 79 in 2017 postsurvey, 90 in matched presurvey, and 55 in matched postsurvey.

Improvement was observed among matched ninth graders (n = 321) regarding their knowledge that accepting a prescription medication from a friend was drug misuse. For Question 93, *P* = .01 (Table 2). After receiving the intervention, among the matched students, we observed an 8% increase in knowledge of identifying addiction as a chronic brain disorder (aOR, 1.08; 95% CI, 0.64–1.83), a 26% increase in understanding of reasons people use drugs (aOR, 1.26; 95% CI, 0.79‒1.98), and a 78% increase in knowledge that nonmedical use is using a prescription medication without a prescription (aOR, 1.78; 95% CI, 0.87–3.6) (Table 2). Knowledge improvement, however, was not significantly higher among ninth graders in the returning schools versus ninth graders in the new schools.

## Implications for Public Health

In our assessment of an opioid misuse education program among ninth- and tenth-grade students in Rhode Island, we found that ninth-grade students had improved knowledge of opioid misuse and improved perceptions of people who use drugs after the education program during the 2017 through 2018 academic year, demonstrating effectiveness of the program. We also found a positive, although nonsignificant, spillover effect among ninth graders from the 3 returning schools. Although a more robust assessment of the spillover mechanism is needed, these findings indicate that an educational awareness and prevention program needs consistent delivery to provide beneficial outcomes.

Our study has several limitations. Although each school administration approved survey implementation, the Rhode Island Department of Education halted it for several months before revoking the approval midstudy. Approval was again obtained after the department’s expanded review of the identical survey; however, critical time lost during the study negatively affected data collection and limited evaluation of spillover effects. We received 1,030 preintervention survey respondents from ninth graders; however, only 415 students completed the postsurvey, and we were only able to match 321 pre- and postrespondents. The preintervention survey had approximately 30% missing answers and the postintervention survey had approximately 20% missing. A low response rate to the postsurvey and missing outcomes limited our ability to detect improvements in knowledge. Another limitation was our use of a fully conditional specification, a semiparametric method that is flexibly suitable for this study. One known theoretical limitation of this study is its high sensitivity to imputing values, and this might have affected our results (13). To avoid this drawback we imputed variables following the order that they appeared in the survey, which was appropriate and matched the monotone pattern of missing data. Additionally, selection bias might have been introduced by student self-reports of earning A or B grades. Students who received good grades might have been more likely to respond to the survey than students who received lower grades.

Future studies could use a randomized design, which might provide a more accurate evaluation of spillover effects of the RAMS-PEER intervention and offer additional insights into the intervention coverage levels needed to change and sustain norms around opioid misuse among adolescents.
